# New-Onset Atrial Fibrillation Is a Risk Factor of Ischemic Stroke in Chronic Obstructive Pulmonary Disease

**DOI:** 10.3390/healthcare10020381

**Published:** 2022-02-17

**Authors:** Chi-Chun Liu, Yu-Hsuan Chen, Yin-Han Chang, Wu-Chien Chien, Hui-Chen Lin, Chun-Gu Cheng, Chun-An Cheng

**Affiliations:** 1 Department of Nursing, Tri-Service General Hospital, National Defense Medical Center, Taipei 11490, Taiwan; vicky122060@gmail.com; 2 Division of Chest Medicine, Department of Internal Medicine, Cheng Hsin General Hospital, Taipei 11220, Taiwan; anemia0829@gmail.com; 3 Department of Psychology, National Taiwan University, Taipei 10621, Taiwan; caalice2003@yahoo.com.tw; 4 Graduate Institute of Life Sciences, National Defense Medical Center, Taipei 11490, Taiwan; chienwu@ndmctsgh.edu.tw; 5 Department of Medical Research, Tri-Service General Hospital, National Defense Medical Center, Taipei 11490, Taiwan; 6 School of Public Health, National Defense Medical Center, Taipei 11490, Taiwan; 7 School of Nursing, College of Nursing, Taipei Medical University, Taipei 11031, Taiwan; ceciliatsgh@gmail.com; 8 Department of Neurology, Tri-Service General Hospital, National Defense Medical Center, Taipei 11490, Taiwan; 9 Department of Emergency Medicine, Taoyuan Armed Forces General Hospital, Taoyuan 32549, Taiwan; 10 Department of Emergency Medicine, Tri-Service General Hospital, National Defense Medical Center, Taipei 11490, Taiwan; 11 Department of Emergency and Critical Medicine, Wan Fang Hospital, Taipei Medical University, Taipei 11696, Taiwan

**Keywords:** new-onset atrial fibrillation, chronic obstructive pulmonary disease, ischemic stroke

## Abstract

Chronic obstructive pulmonary disease (COPD) induces atrial fibrillation (AF) and stroke, and COPD with AF increased ischemic stroke (IS) in a cross-sectional study. Therefore, healthcare providers must be concerned and well-informed about this particular situation. For this study, inpatient data were obtained from the Taiwan National Health Insurance Database in 2010. We identified patients who were hospitalized with COPD (International Classification of Disease, Ninth Revision, Clinical Modification [ICD-9-CM] is 491, 492, and 496). Patients who experienced AF (ICD-9-CM to 427.3) during the same admission or after COPD hospitalization were discharged and defined as new-onset AF. The outcome was IS (ICD-9-CM as 433–437). The factors related to IS after COPD were used for multivariate logistic regression. There were 4177/62,163 (6.72%) patients with incident IS. The risk of IS after COPD hospitalization was shown to have an adjusted odds ratio of 1.749 (95% CI: 1.584–1.93, *p* < 0.001) for patients with new-onset AF. Other factors included advanced age, atherosclerosis factors, comorbidity severity, sepsis and lower-level hospital admission. In conclusion, COPD patients suffering from new-onset AF had an increased incidence of IS in the population observation study. New-onset AF was an omit risk factor for IS in COPD in the Chinese population.

## 1. Introduction

Chronic obstructive pulmonary disease (COPD) is a persistent and progressive airflow limitation. COPD may limit activity, affect quality of life, and lead to disability. The number of patients with COPD has increased to 384 million, and the international prevalence rate is 11.7%. The overall prevalence in men ≥30 years old is 14.3% compared with 7.6% in women [1]. The most important risk factors for COPD are smoking, advanced age and environmental pollution. Acute exacerbation results in the overall severity of the condition, leading to an accelerated decline in lung function with significant mortality.

Stroke is a growing global health problem; ischemic stroke (IS) accounted for 74% of all strokes in the Taiwan registry study [2]. Several comorbidities associated with COPD are independent risk factors for cardiovascular morbidity and mortality. COPD and IS share many of the same risk factors, such as advanced age and smoking, but this does not fully explain their coexistence. In a meta-analysis, patients with COPD were found to have a 30% increased risk of stroke compared with non-COPD patients [3]. The central nervous system and respiratory system are complexly interrelated, and it is hoped that the management of patients with these conditions can occur at the same time, especially in the clinical care setting.

Atrial fibrillation (AF) is the most common persistent heart disease in the world, affecting an estimated 3% of adults. AF is associated with an increased incidence of coronary heart disease, cerebrovascular accidents and other thromboembolism events, chronic congestive heart failure, and low quality of life. Past studies have found that 14% of patients with COPD have AF occurrence. Comparing patients with COPD and no COPD, the AF risk ratio was 1.28 [95% CI (confidence interval): 1.04–1.57], and the risk ratio was 1.99 (95% CI: 1.42–2.79) for frequent exacerbation [4]. Nontraditional risk factors included systemic inflammation, oxidative stress caused by cerebrovascular insufficiency with endothelial dysfunction through arterial stiffness and increased platelet activity. A previous cross-sectional study including 500 patients found a higher incidence of IS in patients with both AF and COPD than in patients with COPD or AF, with adjusted odds ratios (ORs) of 2.85 (95% CI: 1.57–5.16), 1.81 (95% CI: 0.94–3.47) for AF alone and 1.08 (95% CI: 0.58–2.10) for COPD alone [5]. However, the relationship between COPD and AF and IS in the population is unclear.

The aim of our study was to examine whether new-onset AF is a risk factor for IS in patients with COPD. We identified IS risk factors in COPD patients using the National Health Insurance Database (NHIRD) in Taiwan. We aim to identify vulnerable patients and support adequate strategies to reduce the incidence of IS in severe COPD by increasing the awareness of risk factors and highlighting opportunities for improving prevention and treatment.

## 2. Materials and Methods

National Health Insurance, the unique governmental insurance system in Taiwan, has been in practice since 1995 with the inclusion of 99% of the population. The NHIRD contains a health database of Taiwan because Medicare clinic units have demanded this in order to upload coded data to claim insurance payments. The data include each patient’s age, sex, comorbid conditions, IS and COPD codes, and times of events [6].

This study used the inpatient dataset including 5 diagnostics plus 5 operational codes. The duration of the study was from 1 January 2010 to 31 December 2010. The first admission for COPD was retrieved via International Classification of Disease, Ninth Revision; Clinical Modification (ICD-9-CM) codes 491, 492, and 496. AF was determined through the ICD-9-CM code 427.3x. Patients who experienced AF during the same admission or after COPD hospitalization were discharged and defined as new-onset AF. The events of IS were defined as ICD-9-CM codes 433–437. The exclusion criteria were prior IS and prior AF before COPD admission. The comorbid conditions, as assessed by ICD-9-CM codes, included hypertension (401–405), diabetes mellitus (250), coronary heart disease (410–414), congestive heart failure (428), hyperlipidemia (272), chronic kidney disease stages 2–4 (582–584, 588), end-stage renal disease (585,586), sepsis (038, 003.1, 036.1), peripheral artery disease (443), anemia (285), and alcoholism (291,303).

**Figure 1:**
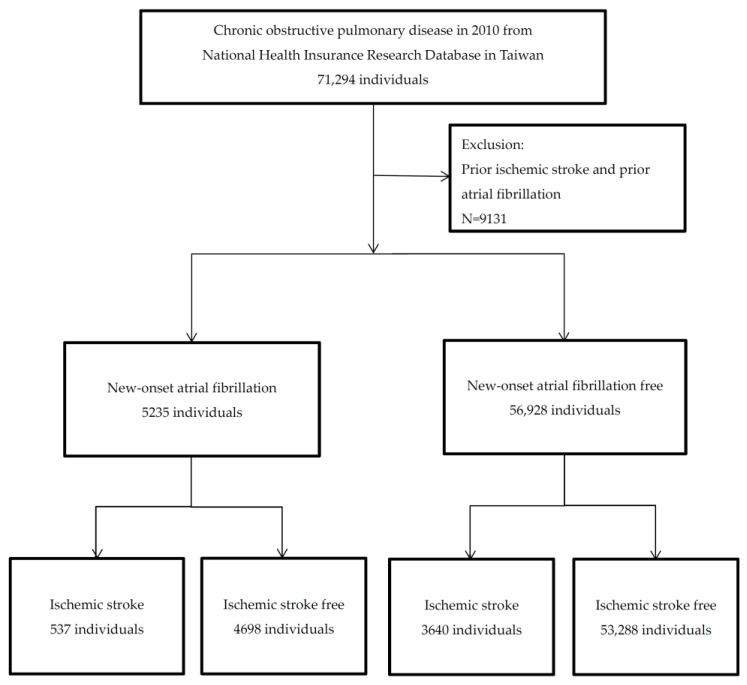
The flowchart of this study.

The Charlson comorbidity index (CCI) is defined as the severity of comorbidities sum calculated from the following scores: age 50–59 years: 1, 60–69 years: 2, 70–79 years: 3, or >80 years: 4; myocardial infarction: 1; congestive heart failure: 1; peripheral arterial disease: 1; stroke: 1; dementia: 1; COPD: 1; connective tissue disease: 1; peptic ulcer disease: 1; liver disease as mild: 1, or moderate or severe: 2; diabetes mellitus as uncomplicated: 1, or end-organ damage: 2; hemiplegia: 2; moderate to severe chronic kidney disease: 2; solid tumors as localized: 2, and metastatic: 6; leukemia: 2; lymphoma: 2; and acquired immunodeficiency syndrome: 6 [7]. Frequent exacerbations of COPD were defined as people who had two or more treated exacerbations during multiple hospitalizations during the study period. The study flowchart is presented in Figure 1. This study was approved by the Tri-Service General Hospital Ethics Institutional Review Board (TSGHIRB: B-I05-11).

The Student’s *t* test was applied for continuous variables, and the chi-square test (X^2^) was used to evaluate categorical factors for differences. The risk of IS in different level hospitals was checked for sensitivity. The risk factors for IS or new-onset AF were identified using a multivariate logistic regression model with forward selection. The statistical significance was set as *p* < 0.05. All statistical analyses were performed using SPSS software version 21 (International Business Machines Company, Armonk, NY, USA).

## 3. Results

There were 62,163 COPD patients evaluated after exclusion of 9131 patients; 5235 (8.42%) had new-onset AF and 4177 (6.72%) had incident IS. A greater proportion of the IS patients had advanced age, higher CCI scores, frequent exacerbations, new-onset AF, hypertension, diabetes mellitus, hyperlipidemia, coronary artery disease, peripheral artery disease, sepsis, local hospital hospitalization and a lower rate of congestive heart failure than the IS-free patients (Table 1).

The risk factors for IS were as follows: advanced age, with an adjusted OR of 1.015 (95% CI: 1.011–1.018, *p* < 0.001); new-onset AF, 1.749 (95% CI: 1.584–1.93, *p* < 0.001); CCI, 1.12 (95% CI: 1.106–1.134, *p* < 0.001); hypertension, 1.323 (95% CI: 1.236–1415, *p* < 0.001); diabetes mellitus, 1.236 (95% CI: 1.147–1.332, *p* < 0.001); hyperlipidemia, 2.229 (95% CI: 1.892–2.626, *p* < 0.001); coronary artery disease, 1.375 (95% CI: 1.275–1.482, *p* < 0.001); sepsis, 1.208 (95% CI: 1.116–1.309, *p* < 0.001); regional hospital admission, 1.132 (95% CI: 1.039–1.235, *p* = 0.005); local hospital admission, 1.574 (95% CI: 1.44–1.721, *p* < 0.001); lower risk with an adjusted OR 0.697 in congestive heart failure (*p* < 0.001), with 0.718 in chronic kidney disease stage 2–4 and with 0.797 in end-stage renal disease compared with chronic kidney disease free (*p* = 0.003 and 0.017) (Table 2). The risk of IS were adjusted OR 2.223 (95% CI: 1.83–2.701, *p* < 0.001) in medical centers, adjusted OR 1.781(95% CI: 1.53–2.072, *p* < 0.001) in region hospitals and adjusted OR 1.42 (95% CI 1.188–1.698, *p* < 0.001) in local hospitals (Table 3).

The increase in new-onset AF among COPD factors included advanced age with an adjusted OR of 1.03 (95% CI: 1.027–1.033, *p* < 0.001); female sex, 1.084 (95% CI: 1.014–1.159, *p* = 0.019); frequent exacerbation, 1.578 (95% CI: 1.486–1.675, *p* < 0.001); coronary artery disease, 1.482 (95% CI: 1.387–1.585, *p* < 0.001); congestive heart failure, 3.586 (95% CI: 3.347–3.841, *p* < 0.001); sepsis, 1.098 (95% CI: 1.021–1.182, *p* = 0.012); regional hospital admission, 1.431 (95% CI: 1.321–1.551, *p* < 0.001); and medical center hospitalization, 1.2 (95% CI: 1.119–1.288, *p* < 0.001) (Table 4).

## 4. Discussion

Our study showed that COPD followed by new-onset AF increased IS occurrence. We explored whether older age and more atherosclerosis risk factors will increase the likelihood of incident IS. Therefore, new-onset AF in COPD is a risk factor of IS incidence. Healthcare providers must be aware of these conditions and aggressively treat lung disease to reduce new-onset AF occurrence to prevent IS.

The most important mechanism in IS was atherosclerosis. Endothelial damage interacts with white blood cells, platelets, and macrophages across the vessel wall. Lipid-rich atherosclerotic plaques attract phagocytic macrophages and eventually rupture and occlude blood vessels [8]. The important risk factors for IS found in multinational studies included hypertension, current smoking habit, waist-to-hip ratio, dietary risk score, regular physical activity, diabetes, hyperlipidemia, alcohol intake, psychosocial stress and depression, and cardiac causes (AF, prior myocardial infarction, rheumatic valvar disease or prosthetic heart disease) [9]. AF is an important factor in cardiac-caused IS. The frequency of AF in the overall hospitalization population was 2.02% (37,504/1,858,112) of Taiwan in 2010.

Approximately 9% of IS, and the most common origin, is caused by AF [10]. Non-AF includes atherosclerotic intracranial stenosis, significant carotid stenosis and complex aortic plaque-associated IS [11]. Hypoxia with hyperventilation in COPD increased sympathetic activity indirectly with a prolonged QTc; moreover, hypercapnia with acidosis can lead to pulmonary hypertension followed by right ventricular hypertrophy and diastolic dysfunction and increase the right atrial diameter. Interestingly, a decrease in lung function in patients with COPD has been found to be inversely associated with AF [12]. Systemic inflammation may play a role in AF originating in the left atrium. Oxidative stress-induced ectopic attack lesions aggravate left ventricular contraction dysfunction, and COPD promotes atrial structure remodeling over a long period of time.

Previous studies have found that frequent COPD deterioration and left atrial size are correlated. The risk of the development of AF in COPD patients with elevated plasma levels of high-sensitivity chronic reaction protein and interleukin 6 is significantly increased, showing a 1.5-fold increase [4]. A previous study found that more risk factors for AF in COPD included age, male sex, hypertension, diabetes, myocardial infarction, heart valve disease, heart failure, obstructive sleep apnea, chronic kidney disease, hyperthyroidism, obesity, heavy alcohol consumption and smoking. Medication treatment for COPD, such as beta agonists shortening atrial refractory periods, anticholinergics inhibiting parasympathetic activity, methylxanthines through depolarization with increased potassium and magnesium excretion, and oral corticosteroids by potassium efflux, might promote a pathophysiological mechanism during the development of AF [13]. Our study found that COPD with new-onset AF occurred in advanced age, with a high frequency of attacks and multiple risk factors. Acute exacerbation of COPD usually lasts for several days; most exacerbations are thought to be caused by infections [14] and are associated with increased systemic inflammation and intense oxidative stress [15]. Sepsis could cause new-onset AF [16], and we found similar results. Patients with COPD admitted to medical centers with multiple complications were more severe than local hospital admissions with incident new-onset AF. Our study showed similar findings in COPD patients. Diabetic patients with poor control could experience polyneuropathy combined with autonomic dysfunction, which reduces the incidence of AF. Hypertension and hyperlipidemia are the claim codes, and appropriate treatment is given to reduce the chances of new-onset AF.

Our study selected hospitalized COPD patients; they had more advanced conditions and a higher mortality rate. We excluded prior AF because of the true risk of IS incidence by new-onset AF, and the lack of a comparison of non-COPD patients may cause a lower risk than a previous study [3,5]. Increased oxidative stress triggers inflammation, deoxyribonucleic acid damage, protein denaturation, and lipid peroxidation [17]. COPD is related to increased arterial stiffness [18]. COPD was associated with a 2.0-fold increase in carotid intimal thickness and a 2.1-fold increase in lipid-rich plaques [19]. Atherosclerosis, assessed for aortic calcification, may also be associated with small vascular changes in COPD [20]. These include anticholinergic and sympathomimetic drugs, macrolides and quinolone antibiotics, which are related to an increased risk of adverse cardiovascular side effects [21,22].

Stroke is an aging disease, and the incidence rate per decade is double after 55 years of age [23]. Our study found a 1.5% increase in IS risk for each additional year. Hyperlipidemia increased IS by 2.40 times, and our results were similar. COPD hospitalization due to infection was followed by sepsis. Past studies found that sepsis increases IS risk by 1.75 times within six months [24], and our study found that sepsis increases IS with a milder risk. The potential reasons were that COPD itself increased IS by competing for risk, and sepsis caused a 1.5-fold risk of death in our study. A previous sepsis study found that IS was more common in local hospitals, with a 1.25-fold higher risk [16]. Similar findings were found for higher IS risk in lower-level hospitals in our study. The potential reason may be that lower-level hospitals have inadequate medical facilities and inappropriate preventive strategies. There were fewer doctors and nurses in regional and local hospitals, they suffered from heavy load and did not follow guidelines closely induced higher events. The education of healthcare providers in lower-level hospitals can improve the healthcare quality. The self-reported history of hypertension in multi-country studies showed an increase in IS by 2.37 times, and an increase in diabetes by 1.60 times compared with IS. The chance of the cardiac cause increased by 2.74 [9]. Our study found a milder risk of hypertension, diabetes, and coronary heart disease than past studies, potentially because COPD itself increases IS, and the risk is reduced by this interaction.

The relationship between sex and stroke depended on age. The stroke rate is higher in young females than in males. In contrast, in the older generation, males have a higher risk [25]. The prevalence of COPD was predominant in males (approximately 75%) in our study, and women with AF had IS incidents in a previous study [26]. Female COPD patients had more frequent new-onset AF in our study. These causes balanced the risk of IS, and sex did not affect IS in COPD.

Previous studies have found a 43% increase in the risk of IS in the first 3 days. The peak risk of IS occurred at 4 days after an acute exacerbation of COPD, with an 80% increase in risk (greater than or equal to two times). There was no significantly increased IS risk (IRR = 1.17, 95% CI: 0.83–1.67) after 1 month in the chronic period [27]. The potential reasons were frequent medical help and access to a more powerful risk reduction strategy with fewer chances of inflammation [28]. Our study showed similar results. A possible additional reason is that frequent exacerbations carry a 3.5-fold risk of death and compete with this risk. The risk of stroke in COPD was significantly higher in patients due to embolization and hypoperfusion with right heart failure than in patients without right heart failure [29]. However, our study showed a lower risk of IS with a lower proportion of congestive heart failure. A prevalence rate study showed that more subjects with chronic kidney disease suffered from IS than subjects without chronic kidney disease [30]. In our study, chronic kidney disease did not increase the incidence of IS in COPD because the risk of death was twice that caused by chronic kidney disease stages 2–4 and end-stage renal disease in COPD, which also competes with the risk of IS (Supplementary Material Table S1: Factors of death in chronic obstructive pulmonary disease with multivariate logistic regression). Patients with COPD and stroke share conditions including diabetes, hypertension and peripheral arterial disease [29]. However, because of the low prevalence, peripheral arterial disease did not increase IS. The mortality of COPD was relatively higher than no-COPD. The new-onset AF induced IS will be given anticoagulant therapy by guidelines reducing the short-term mortality.

Administration of antiplatelet therapy is associated with IS prevention [31]. Beta blockers induce fewer myocardial infarctions than calcium blockers, and beta blockers have fewer cardiovascular hospitalizations than digoxin [32]. In patients with COPD compared with non-COPD patients, the use of beta blockers at discharge significantly reduced 1-year mortality after discharge (SHR 0.66, 95% CI: 0.53–0.83) [33]. However, past studies with congestive heart failure combined with COPD have found that the presence of COPD significantly reduces the prescription of beta blockers at discharge. Educating physicians about selective beta blockers for COPD can reduce AF and thus reduce IS [34]. The ARISTOTLE study found a higher mortality rate for AF and COPD, and the use of anticoagulants effectively reduced the incidence of stroke or systemic embolism with COPD compared with non-COPD [35].

Systemic inflammation in COPD requires further research to examine these therapeutic interventions. A recent meta-analysis confirmed that statins were associated with a significant reduction in the risk of myocardial infarction but lacked evidence for stroke prevention [36]. As recommended in the Global Initiative for Chronic Obstructive Lung Disease (GOLD) guidelines, severe COPD acute aggravation treatment with high doses of systemic corticosteroids is associated with an increased risk of AF, while low-dose corticosteroid reduction in chronic reaction protein has previously been shown to significantly prevent AF recurrence after cardiac resuscitation [37,38]. The meta-analysis found inconclusive results with colchicine for stroke prevention [39]. In a recent study, colchicine provided an anti-inflammatory effect and reduced IS attack after acute myocardial infarction [40]. Anti-inflammatory therapy in COPD to prevent IS needs future evaluation. To enhance inadequate medical facilities in local and regional hospitals, it is necessary to educate doctors in hospitals that COPD with new-onset AF is a possible risk factor for IS and strengthen care and appropriate treatment to reduce IS.

There were some limitations in our study. First, the NHIRD claims database does not have information on smoking, drinking and body mass index. In previous COPD and stroke studies, the risk assessment after adjusting for smoking was reduced by only approximately 10% [41]. In addition, this suggests that other pathogenic effects of COPD are beyond the harm of smoking itself. Second, our study did not distinguish between paroxysmal and persistent AF and did not provide vital capacity data in all subjects. However, some potential confounding factors have not been considered, including valvar heart disease, hyperthyroidism and obstructive sleep apnea, due to their lower prevalence in hospitalization. Third, our study of patients hospitalized for IS could not identify the severity of IS, and computer tomography or magnetic resonance imaging scans were unavailable. Thus, imaging studies are needed to complement this deficiency in the future. Fourth, the population with COPD and AF but without ischemic stroke is not hospitalized and could underestimate the problem. In the data of this study from several years ago, the healthcare providers paid more attention to the AF incidence in COPD and new oral anticoagulants therapy for high risk AF patients to prevent IS that possibly reduced the IS occurrence in recent years.

## 5. Conclusions

This study noted the increased risk of IS with COPD plus new-onset AF in Taiwan. After adjusting for the associative confounding factors, new-onset AF increased the risk of IS in patients with COPD by 75%. Healthcare providers should alert this hurdle and focus on aggressive COPD management and infection control strategies to reduce new-onset AF occurrence and promote IS prevention.

## Figures and Tables

**Table 1:** Baseline characteristics of patients by ischemic stroke status in chronic obstructive pulmonary disease.

	Ischemic Stroke Free (57,986)	Ischemic Stroke (4177)	*p*
Age	75.91 ± 12.47	77.93 ± 10.21	<0.001 *
Sex (male)	43,651 (75.28%)	3120 (74.69%)	0.404
Charlson comorbidity index	2.05 ± 1.96	2.49 ± 1.51	<0.001 *
Exacerbation frequency	1.81 ± 1.68	1.82 ± 1.49	0.491
Frequent exacerbation (>2)	22,223 (38.32%)	1730 (41.42%)	<0.001 *
New-onset atrial fibrillation	3640 (6.28%)	537 (12.86%)	<0.001 *
Hypertension	18,190 (31.37%)	1572 (37.63%)	<0.001 *
Diabetes mellitus	11,297 (19.48%)	1041 (24.92%)	<0.001 *
Hyperlipidemia	1240 (2.14%)	181 (4.33%)	<0.001 *
Coronary artery disease	10,896 (18.79%)	1045 (25.01%)	<0.001 *
Congestive heart failure	7196 (12.41%)	448 (10.73%)	0.001 *
Anemia	2127 (3.67%)	138 (3.3%)	0.249
Alcoholism	607 (1.05%)	35 (0.84%)	0.236
Peripheral artery disease	772 (1.33%)	73 (1.75%)	0.038 *
Chronic kidney disease			0.078
Chronic kidney disease stages 2–4	1522 (2.62%)	87 (2.08%)	
End-stage renal disease	1899 (3.27%)	128 (3.06%)	
Sepsis	12,987 (19.60%)	1126 (22.42%)	<0.001 *
Hospital type			<0.001 *
Medical center	13,689 (23.61%)	838 (20.06%)	
Regional hospital	25,953 (44.76%)	1703 (40.77%)	
Local hospital	18,344 (31.63%)	1636 (39.17%)	

* *p* < 0.05.

**Table 2:** Factors related to ischemic stroke in chronic obstructive pulmonary disease with multivariate logistic regression.

Factors	Adjusted Odds Ratio (95% Confidence Interval)	*p*
Age	1.015 (1.011–1.0185)	<0.001 *
Charlson comorbidity index	1.12 (1.106–1.134)	<0.001 *
New-onset atrial fibrillation	1.749 (1.584–1.93)	<0.001 *
Hypertension	1.323 (1.236–1.415)	<0.001 *
Diabetes mellitus	1.236 (1.147–1.332)	<0.001 *
Hyperlipidemia	2.229 (1.892–2.626)	<0.001 *
Coronary artery disease	1.375 (1.275–1.482)	<0.001 *
Congestive heart failure	0.697 (0.628–0.774)	<0.001 *
Chronic kidney disease		
Chronic kidney disease free	Reference	
Chronic kidney disease stages 2–4	0.718 (0.576–0.895)	0.003 *
End-stage renal disease	0.797 (0.663–0.963)	0.017 *
Sepsis	1.208 (1.116–1.309)	<0.001*
Hospital type		
Medical center	Reference	
Regional hospital	1.132 (1.039–1.235)	0.005 *
Local hospital	1.574 (1.44–1.721)	<0.001 *

* *p* < 0.05.

**Table 3:** The risk factors for ischemic stroke according to different hospital levels.

	Medical Centers		Regional Hospitals		Local Hospitals	
Factors	Odds Ratio (95% Confidence Interval)	*p*	Odds Ratio (95% Confidence Interval)	*p*	Odds Ratio (95% Confidence Interval)	*p*
Age	1.024 (1.016–1.031)	<0.001 *	1.017 (1.012–1.022)	<0.001 *	1.008 (1.003–1.012)	0.001 *
Charlson comorbidity index	1.06 (1.034–1.086)	<0.001 *	1.111 (1.091–1.132)	<0.001 *	1.35 (1.3074–1.395)	<0.001 *
New-onset atrial fibrillation	2.223 (1.83–2.701)	<0.001 *	1.781 (1.53–2.072)	<0.001 *	1.42 (1.188–1.698)	<0.001 *
Hypertension	1.178 (1.013–1.371)	0.033 *	1.389 (1.253–1.541)	<0.001 *	1.366 (1.224–1.524)	<0.001 *
Diabetes mellitus	1.252 (1.052–1.489)	0.011 *	1.191 (1.057–1.341)	0.004 *		
Hyperlipidemia	2.559 (1.9–3.447)	<0.001 *	2.846 (2.264–3.577)	<0.001 *		
Coronary artery disease	1.196 (1.013–1.371)	0.044 *	1.208 (1.069–1.365)	<0.001 *	1.659 (1.478–1.863)	<0.001 *
Sepsis	1.311 (1.086–1.582)	0.005 *			1.274 (1.132–1.433)	<0.001 *
Congestive heart failure	0.73 (0.73–0.587)	0.005 *	0.756 (0.648–0.862)	<0.001 *		
Chronic kidney disease free			Reference		Reference	
Chronic kidney disease				0.047 *		<0.001 *
Chronic kidney disease stage 2–4			0.731 (0.509–1.049)	0.089	0.483 (0.326–0.714)	<0.001 *
End-stage renal disease			0.745 (0.544–1.02)	0.066	0.677 (0.503–0.911)	0.01 *
Anemia			0.632 (0.431–0.927)	0.019*		

* *p* < 0.05.

**Table 4:** Factors of new-onset atrial fibrillation in chronic obstructive pulmonary disease with multivariate logistic regression.

Factors	Adjusted Odds Ratio (95% Confidence Interval)	*p*
Age	1.03 (1.027–1.033)	<0.001 *
Gender (female)	1.084 (1.014–1.159)	0.019 *
Charlson comorbidity index	0.903 (0.883–0.923)	<0.001 *
Frequent exacerbation	1.578 (1.486–1.675)	<0.001 *
Hypertension	0.911 (0.854–0.971)	0.004 *
Diabetes mellitus	0.894 (0.824–0.969)	0.007 *
Hyperlipidemia	0.724 (0.572–0.915)	0.007 *
Coronary artery disease	1.482 (1.387–1.585)	<0.001 *
Congestive heart failure	3.586 (3.347–3.841)	<0.001 *
Anemia	0.5981 (0.494–0.725)	<0.001 *
Sepsis	1.098 (1.021–1.182)	0.012 *
Hospital type		
Local hospital	Reference	
Regional hospital	1.431 (1.321–1.551)	<0.001 *
`Medical center	1.2 (1.119–1.288)	<0.001 *

* *p* < 0.05.

## Data Availability

The datasets used in the current study are available from the corresponding author on reasonable request.

## Supplementary Materials

The following supporting information can be downloaded at: https://www.mdpi.com/article/10.3390/healthcare10020381/s1, Table S1: Factors of death in chronic obstructive pulmonary disease with multivariate logistic regression.
