# Supplementary material for: New-Onset Atrial Fibrillation Is a Risk Factor of Ischemic Stroke in Chronic Obstructive Pulmonary Disease
Source: Healthcare (Basel). 2022 Feb 17;10(2):381. doi: 10.3390/healthcare10020381 (PMC8871767; doi:10.3390/healthcare10020381)
Supplement: Supplementary file 1 [file healthcare-10-00381-s001.zip › healthcare-1603412-supplementary.pdf]

Table S1. Factors of death in chronic obstructive pulmonary disease with multivariate logistic regression.

Factors	Adjusted odds ratio (95% confidence interval)	*p*
Age	1.048 (1.043-1.052)	<0.001*
Sex (Female)	1.221 (1.115-1.337)	0.002*
Charlson Comorbidity index	1.097 (1.181-1.113)	<0.001*
Frequent exacerbation	3.541 (3.248-3.86)	<0.001*
New-onset atrial fibrillation	0.702 (0.606-0.812)	<0.001*
Hypertension	0.363 (0.324-0.407)	<0.001*
Diabetes mellitus	0.685 (0.612-0.718)	<0.001*
Hyperlipidemia	0.178 (0.074-0.43)	<0.001*
Coronary artery disease	0.519 (0.46-0.586)	<0.001*
Anemia	0.68 (0.545-0.847)	<0.001*
Chronic kidney disease		
Chronic kidney disease free	Reference	
Chronic kidney disease stages 2-4	2.105 (1.766-2.51)	<0.001*
End-stage renal disease	1.885 (1.577-2.252)	<0.001*
Sepsis	1.499 (1.374-1.635)	<0.001*
Hospital type		
Medical center	Reference	
Regional hospital	0.903 (0.813-1.002)	0.055
Local hospital	1.126 (1.01-1.255)	0.032*

*P<0.05
